# External Validation of an Ensemble Model for Automated Mammography Interpretation by Artificial Intelligence

**DOI:** 10.1001/jamanetworkopen.2022.42343

**Published:** 2022-11-21

**Authors:** William Hsu, Daniel S. Hippe, Noor Nakhaei, Pin-Chieh Wang, Bing Zhu, Nathan Siu, Mehmet Eren Ahsen, William Lotter, A. Gregory Sorensen, Arash Naeim, Diana S. M. Buist, Thomas Schaffter, Justin Guinney, Joann G. Elmore, Christoph I. Lee

**Affiliations:** 1 Medical and Imaging Informatics, Department of Radiological Sciences, David Geffen School of Medicine at University California, Los Angeles; 2 Clinical Research Division, Fred Hutchinson Cancer Center, Seattle, Washington; 3 Department of Medicine, David Geffen School of Medicine at University California, Los Angeles; 4 Medical Informatics Home Area, Graduate Programs in Biosciences, David Geffen School of Medicine at University California, Los Angeles, Los Angeles, California; 5 Gies College of Business, University of Illinois at Urbana-Champaign; 6 DeepHealth, RadNet AI Solutions, Cambridge, Massachusetts; 7 Center for Systematic, Measurable, Actionable, Resilient, and Technology-driven Health, Clinical and Translational Science Institute, David Geffen School of Medicine at University California, Los Angeles; 8 Kaiser Permanente Washington Health Research Institute, Seattle, Washington; 9 Computational Oncology, Sage Bionetworks, Seattle, Washington; 10 Tempus Labs, Chicago, Illinois; 11 Department of Radiology, University of Washington School of Medicine, Seattle; 12 Department of Health Services, University of Washington School of Public Health, Seattle; 13 Hutchinson Institute for Cancer Outcomes Research, Fred Hutchinson Cancer Center, Seattle, Washington

## Abstract

**Importance:**

With a shortfall in fellowship-trained breast radiologists, mammography screening programs are looking toward artificial intelligence (AI) to increase efficiency and diagnostic accuracy. External validation studies provide an initial assessment of how promising AI algorithms perform in different practice settings.

**Objective:**

To externally validate an ensemble deep-learning model using data from a high-volume, distributed screening program of an academic health system with a diverse patient population.

**Design, Setting, and Participants:**

In this diagnostic study, an ensemble learning method, which reweights outputs of the 11 highest-performing individual AI models from the Digital Mammography Dialogue on Reverse Engineering Assessment and Methods (DREAM) Mammography Challenge, was used to predict the cancer status of an individual using a standard set of screening mammography images. This study was conducted using retrospective patient data collected between 2010 and 2020 from women aged 40 years and older who underwent a routine breast screening examination and participated in the Athena Breast Health Network at the University of California, Los Angeles (UCLA).

**Main Outcomes and Measures:**

Performance of the challenge ensemble method (CEM) and the CEM combined with radiologist assessment (CEM+R) were compared with diagnosed ductal carcinoma in situ and invasive cancers within a year of the screening examination using performance metrics, such as sensitivity, specificity, and area under the receiver operating characteristic curve (AUROC).

**Results:**

Evaluated on 37 317 examinations from 26 817 women (mean [SD] age, 58.4 [11.5] years), individual model AUROC estimates ranged from 0.77 (95% CI, 0.75-0.79) to 0.83 (95% CI, 0.81-0.85). The CEM model achieved an AUROC of 0.85 (95% CI, 0.84-0.87) in the UCLA cohort, lower than the performance achieved in the Kaiser Permanente Washington (AUROC, 0.90) and Karolinska Institute (AUROC, 0.92) cohorts. The CEM+R model achieved a sensitivity (0.813 [95% CI, 0.781-0.843] vs 0.826 [95% CI, 0.795-0.856]; *P* = .20) and specificity (0.925 [95% CI, 0.916-0.934] vs 0.930 [95% CI, 0.929-0.932]; *P* = .18) similar to the radiologist performance. The CEM+R model had significantly lower sensitivity (0.596 [95% CI, 0.466-0.717] vs 0.850 [95% CI, 0.766-0.923]; *P* < .001) and specificity (0.803 [95% CI, 0.734-0.861] vs 0.945 [95% CI, 0.936-0.954]; *P* < .001) than the radiologist in women with a prior history of breast cancer and Hispanic women (0.894 [95% CI, 0.873-0.910] vs 0.926 [95% CI, 0.919-0.933]; *P* = .004).

**Conclusions and Relevance:**

This study found that the high performance of an ensemble deep-learning model for automated screening mammography interpretation did not generalize to a more diverse screening cohort, suggesting that the model experienced underspecification. This study suggests the need for model transparency and fine-tuning of AI models for specific target populations prior to their clinical adoption.

## Key Points

**Question:**

Will a high-performing ensemble of artificial intelligence (AI) models for automated interpretation of screening mammography generalize to a diverse population?

**Findings:**

In this diagnostic study using 37 317 examinations from 26 817 women seen at a geographically distributed screening program, a previously validated ensemble model had a decline in performance compared with its reported performance in other, more homogeneous cohorts. When combined with a radiologist assessment, ensemble performance was similar to that of the radiologist, but worse performance was noted in subgroups, particularly Hispanic women and women with a personal history of breast cancer.

**Meaning:**

These findings suggest that AI models, including those trained on large data sets or constructed using ensemble methods, may be at risk of underspecification and poor generalizability.

## Teaser

This diagnostic study evaluates an ensemble artificial intelligence model for automated interpretation of screening mammography in a diverse population.

## Introduction

Advances in artificial intelligence (AI) and machine learning (ML) have accelerated the demand for adopting such technologies in clinical practice. The number of AI and ML approaches for screening mammography classification has dramatically increased given the need for automated triaging and diagnostic tools to manage the high volume of breast screening examinations, shortfall in fellowship-trained breast radiologists, and opportunity for commercialization in this space.^1,2,3,4^ As of March 2022, the Food and Drug Administration has cleared more than 240 radiology AI algorithms,^5^ of which 11 characterize breast lesions on mammography.^6^ The need for a clear understanding of when these algorithms do and do not perform well in the target cohort is an important component of their successful adoption into clinical practice. Historically, most AI studies reported model performance on a set of test cases drawn from the same patient population used to train the model. However, internal validation may not distinguish between equivalently performing models that have learned the correct representation for the problem or are predicated on confounding factors, a situation called underspecification.^7^ External validation using an independent (ie, not identically distributed) population is a critical step to identify models that are at risk of underspecification and ensure the generalizability of a model before clinical adoption.^8^

To date, the largest crowdsourced effort in deep learning and mammography was the Digital Mammography Dialogue on Reverse Engineering Assessment and Methods (DREAM) Challenge,^9,10^ which used 144 231 screening mammograms from Kaiser Permanente Washington (KPW) for algorithm training and internal validation. The final ensemble model was associated with improved overall diagnostic accuracy in combination with radiologist assessment. The ensemble algorithm demonstrated similar performance in a Swedish population, the Karolinska Institute (KI), used for external validation. KPW and KI screening cohorts were composed heavily of White women. The DREAM Challenge ensemble algorithm has yet to be externally validated on a more diverse US screening population, to our knowledge.

Our study objective was to evaluate the performance of the published challenge ensemble method (CEM) from the DREAM Challenge, which incorporated predictions from the 11 top-performing models, using an independent, diverse US screening population. The CEM model is publicly available as open-source software, allowing others to evaluate the algorithm on their local data sets. We evaluated the performance of the CEM against original radiologist reader performance and the performance of the CEM and radiologist combined (CEM+R) in this new, more diverse screening population.

## Methods

### Study Population

This diagnostic study was conducted under a waiver of consent according to 45 CFR §46.116^11^ with approval granted by the University of California, Los Angeles (UCLA) institutional review board. Our study followed the Transparent Reporting of a Multivariable Prediction Model for Individual Prognosis or Diagnosis (TRIPOD) reporting guideline.

We used clinical, imaging, and cancer outcomes data collected as part of the Athena Breast Health Network,^12^ an observational study conducted across breast screening programs at 5 University of California medical centers, including UCLA Health. Women who arrived at an outpatient imaging center for a mammographic or ultrasound breast imaging examination (screening or diagnostic) completed an electronic or hard copy survey related to their health history, lifestyle behaviors, and family history of cancer. The entire UCLA Athena population included 49 244 women who completed 89 881 surveys between December 1, 2010, and October 31, 2015. Imaging examinations and a Breast Imaging Reporting and Data System (BI-RADS) assessment provided by a single radiologist for these women were obtained during the study period and an additional 4 years after accrual, from December 2010 to December 2019. This analysis focused on 2-dimensional screening mammography to match what was used in the original DREAM Mammography Challenge. Breast cancer diagnoses made between December 2010 and December 2020 were obtained from an institutional registry populated with data from hospitals across Southern California. All examinations from the UCLA cohort were acquired using mammography equipment from Hologic, which was similar to methods used in the KPW and KI cohorts. A detailed description of the UCLA cohort and data collected on each individual is provided in eAppendix 1 in the Supplement. The patient selection process is summarized in eFigure 1 in the Supplement, and eTable 1 and eTable 2 in the Supplement list all clinical variables obtained and their level of missingness. See eTable 3 in the Supplement for a list of how individual diagnostic codes obtained from the institutional registry were categorized as ductal carcinoma in situ (DCIS) or invasive cancer.

Examinations were partitioned into 4 groups: true negatives (TNs; consecutive BI-RADS 1 and 2 annual screening examinations with no cancer diagnosis between examinations), false positives (FPs; BI-RADS 0 and no cancer diagnosis within 12 months), true positives (TPs; BI-RADS 0 and cancer diagnosis within 12 months), and false negatives (FNs; BI-RADS 1 and 2 and cancer diagnosis within 12 months). After excluding examinations that could not be downloaded from the picture archiving and communication system (PACS), did not have a standard set of screening images, or were missing clinical data required to execute the model, examinations were randomly sampled from each group to be included in the analysis. In this sampled subset of 37 317 examinations from 26 817 women, cancers and radiologist FPs were oversampled relative to their proportions in the full UCLA cohort to maintain large sample sizes for those important groups, but the inverse probability of sampling weights was used in the analysis so estimates would reflect the proportions of TPs, FNs, FPs, and TNs of the complete cohort (eAppendix 2 in the Supplement; a breakdown of the analyzed subset is shown in eFigure 2 in the Supplement). TN examinations (112 598 of 121 753 examinations total) were undersampled, while FP, TP, and FN examinations were oversampled. The following proportions of number of examinations in the analyzed subset among the number of examinations in the full cohort for these groups were included in the analysis: 33 267 of 112 598 TN examinations (0.295), 3474 of 8432 FP examinations (0.412), 465 of 597 TP examinations (0.779), and 111 of 126 FN examinations (0.881). Weights for these 4 groups were calculated as the inverse of these proportions and are summarized in eTable 4 in the Supplement. Outcomes in target metrics associated with inverse probability are illustrated in eFigure 3 in the Supplement. These weights were used in the statistical analysis so that performance estimates would be representative of the original UCLA cohort of 121 573 examinations.

### Model Execution

The CEM comprises 11 models contributed by the top 6 performing competitive phase teams in the DREAM Mammography Challenge (eAppendix 3 in the Supplement). Each model was treated as a black box given that no modifications were made to the algorithms, which were trained on the KPW data set, before running them on the UCLA data set. Each model generated a confidence score between 0 and 1, reflecting the likelihood of cancer for each side of the breast. The CEM used confidence score outputs from each model as inputs, reweighting them and outputting a combined score.^9^ A modified CEM with radiologist suspicion (CEM+R) was developed, producing an additional input of a binarized overall BI-RADS score provided by the original interpreting radiologist at the examination level. Experiments were performed in a cloud-based environment (Amazon Web Services) using 3 instances running in parallel with a shared file share (Amazon S3 bucket) that hosted all imaging data (eAppendix 4, eFigure 4, and eAppendix 5 in the Supplement).

### Statistical Analysis

The performance of CEM, CEM+R, and radiologists for detecting cancer was summarized using metrics based on the frequency of TPs, FPs, TNs, and FNs. CEM and CEM+R results were considered positive when their risk score exceeded a given threshold, while a radiologist BI-RADS score of 0, 3, 4, or 5 for a screening examination was considered positive. Performance metrics were weighted using inverse probability of selection weights to represent the full UCLA cohort described in eAppendix 2 in the Supplement. Calibration performance was summarized using calibration curves and the calibration intercept and slope.^13^

Primary performance metrics of individual models, CEM, CEM+R, and radiologists were area under the receiver operating characteristic curve (AUROC), sensitivity, and specificity. Secondary performance metrics included positive predictive value (defined as TP/[TP + FP]), abnormal interpretation rate (defined as [TP + FP]/N), cancer detection rate (defined as TP/N), and FN rate (defined as FP/N, where N represents the total number of screening examinations). Threshold-dependent performance metrics of CEM and CEM+R were estimated at thresholds selected to match radiologist sensitivity or specificity estimated from the same sample. AUROC, calibration intercept, and calibration slope were also estimated within subgroups defined by cancer type (DCIS or invasive), age, self-reported race and ethnicity, mammographic breast density, and personal history of breast cancer as part of exploratory subgroup analysis. Survey options for race and ethnicity were American Indian or Alaska Native, Asian, Black, Hawaiian or Pacific Islander, Hispanic, White, mixed, other, and missing (eTable 1 in the Supplement). In our analysis, we used other to include the original survey options American Indian and Alaska Native, Hawaiian and Pacific Islander, mixed, and other. Race and ethnicity were evaluated because they are known factors associated with breast cancer risk. We assessed whether the CEM+R model had differences in sensitivity, specificity, and AUROC by race and ethnicity subgroup, which may indicate a lack of representation of specific racial and ethnic subgroups when training the model.

Performance metric CIs were calculated using nonparametric bootstrap with resampling stratified by TN, FP, TP, and FN groups. We resampled at the patient level rather than examination level to account for the nonindependence of multiple examinations of the same women. Nonparametric bootstrap was also used to calculate CIs and *P* values to compare performance metrics among CEM, CEM+R, radiologists, and patient subgroups. *P* values were 2-sided, and statistical significance was defined as *P* < .05. Statistical analyses were conducted using R statistical software version 4.0 (R Project for Statistical Computing), Python programming language version 3.8 (Python Software Foundation), and the scikit-learn library version 1.0.2 (scikit-learn Developers). Weighted ROC and precision recall curves were estimated using the PRROC package in R version 1.3.1 (Jan Grau and Jens Keilwagen).^14^

## Results

### Comparisons Between UCLA, KPW, and KI Cohorts

Table 1 summarizes characteristics of the UCLA cohort (target), which differed from those of the KPW (development) and KI (external) cohorts; eTable 5 in the Supplement summarizes the differences across all cohorts. The KPW cohort had 144 231 examinations from 85 580 women, of whom 952 women (1.1%) were positive for breast cancer; among them, 697 women (73.2%) had invasive breast cancer. The KI cohort had 166 578 examinations from 68 008 women, of whom 780 women (1.1%) were cancer positive; among them, 681 women (87.3%) had invasive cancer. The UCLA cohort had 121 753 examinations from 41 343 women, of whom 723 women (1.7%) were cancer positive; among them, 567 women (79.4%) had invasive cancer. The UCLA cohort had a higher percentage of cancers at the patient level than the KPW and KI cohorts (714 women [1.7%] vs 952 women [1.1%] and 780 women [1.1%], respectively) (eTable 5 in the Supplement).^9^ Of 26 817 women in the analyzed subset, 573 women (2.1%) had a cancer diagnosis. Among these women, there were 37 317 examinations (mean [SD] age, 58.4 [11.5] years; 3338 Asian [9.7%], 2972 Black [8.6%], 3699 Hispanic [10.6%], 20 602 White [59.3%], and 4093 other race or ethnicity [11.8%] among 34 754 examinations with race and ethnicity data), of which 576 of 37 317 examinations (1.5%) were cancer positive at the examination level. After applying inverse probability weights, the cancer positive rate was 0.6%, the same as the full UCLA cohort (723 of 121 753 examinations [0.6%]) at the examination level.

**Table 1.** Patient and Examination Characteristics

Characteristic	No. (%)		
	Full cohort, unweighted	Analyzed subset^a^	
		Unweighted, No. (%)	Weighted, %
**Women**			
Total, No.	41 343	26 817	NA
Diagnosed with BC within 12 mo of mammogram	714 (1.7)	573 (2.1)	NA
**Screening examinations**			
Total, No.	121 753	37 317	NA
BI-RADS category			
0	8493 (6.9)	3809 (10.2)	NA
1	46 093 (37.9)	14 063 (37.7)	NA
2	66 632 (54.7)	19 315 (51.8)	NA
3, 4, or 5	535 (0.4)	130 (0.3)	
BC diagnosed within 12 mo	723 (0.6)	576 (1.5)	0.6
Invasive BC	576 (79.7)	462 (80.2)	79.9
DCIS	147 (20.3)	114 (19.8)	20.1
No BC diagnosed within 12 mo	121 030 (99.4)	36 741 (98.5)	99.4
Age, mean (SD), y	58.9 (11.6)	58.4 (11.5)	NA
BMI, mean (SD)	28.4 (6.9)	29.0 (7.0)	NA
Personal history of breast cancer^b^	3751 (6.1)	1864 (6.1)	6.1
Race and ethnicity^c^			
Asian	10 913 (10.3)	3338 (9.7)	9.8
Black	9558 (9.0)	2972 (8.6)	8.5
Hispanic	11 449 (10.8)	3699 (10.6)	10.6
White	61 703 (58.3)	20 602 (59.3)	59.3
Other^d^	12 254 (11.6)	4093 (11.8)	11.7

Abbreviations: BC, breast cancer; BI-RADS, Breast Imaging Reporting and Data System; BMI, body mass index (calculated as weight in kilograms divided by height in meters squared); DCIS, ductal carcinoma in situ; NA, not applicable.

^a^ In the analyzed subset of the University of California, Los Angeles (UCLA), cohort, cancer and radiologist false positives were oversampled to maintain large sample sizes; inverse probability of selection weights were used so that weighted means would reflect values from the full UCLA cohort.

^b^ Personal history of breast cancer was available in 61 278 examinations (50.3%) in the full cohort and 30 519 examinations (81.7%) in the analyzed subset.

^c^ Race and ethnicity were available in 105 877/121 753 examinations (86.9%) in the full cohort and 34 754 examinations (93.1%) in the analyzed subset.

^d^ Other included American Indian or Alaska Native, Native Hawaiian or other Pacific Islander, multiple races, or some other race.

### Radiologist Performance

Of the full UCLA cohort, there were 597 TPs, 126 FNs, 8432 FPs, and 112 598 TNs. Radiologist sensitivity and specificity were 0.826 (95% CI, 0.798-0.853) and 0.930 (95% CI, 0.929-0.932), respectively. In the analyzed subset, there were 465 TPs, 111 FNs, 3474 FPs, and 33 267 TNs. The weighted estimates of radiologist sensitivity and specificity in the analyzed subset were similar to those based on the full cohort: 0.826 (95% CI, 0.795-0.856) and 0.930 (95% CI, 0.929-0.932), respectively.

### Individual Model Performance

**Figure 1.**
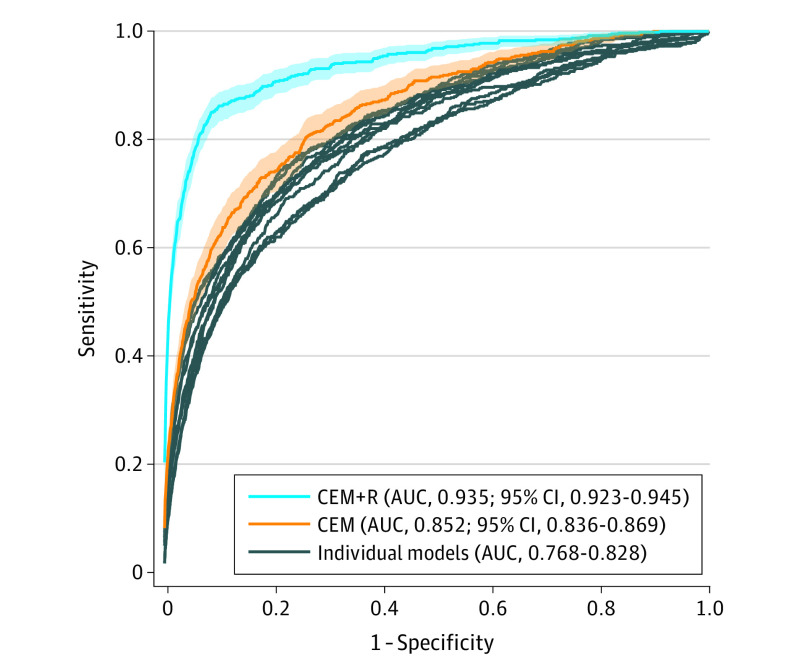
Receiver Operating Characteristic Curves AUC indicates area under the curve; CEM, challenge ensemble method; CEM+R, challenge ensemble method plus radiologist; shaded areas, 95% CIs.

**Figure 2.**
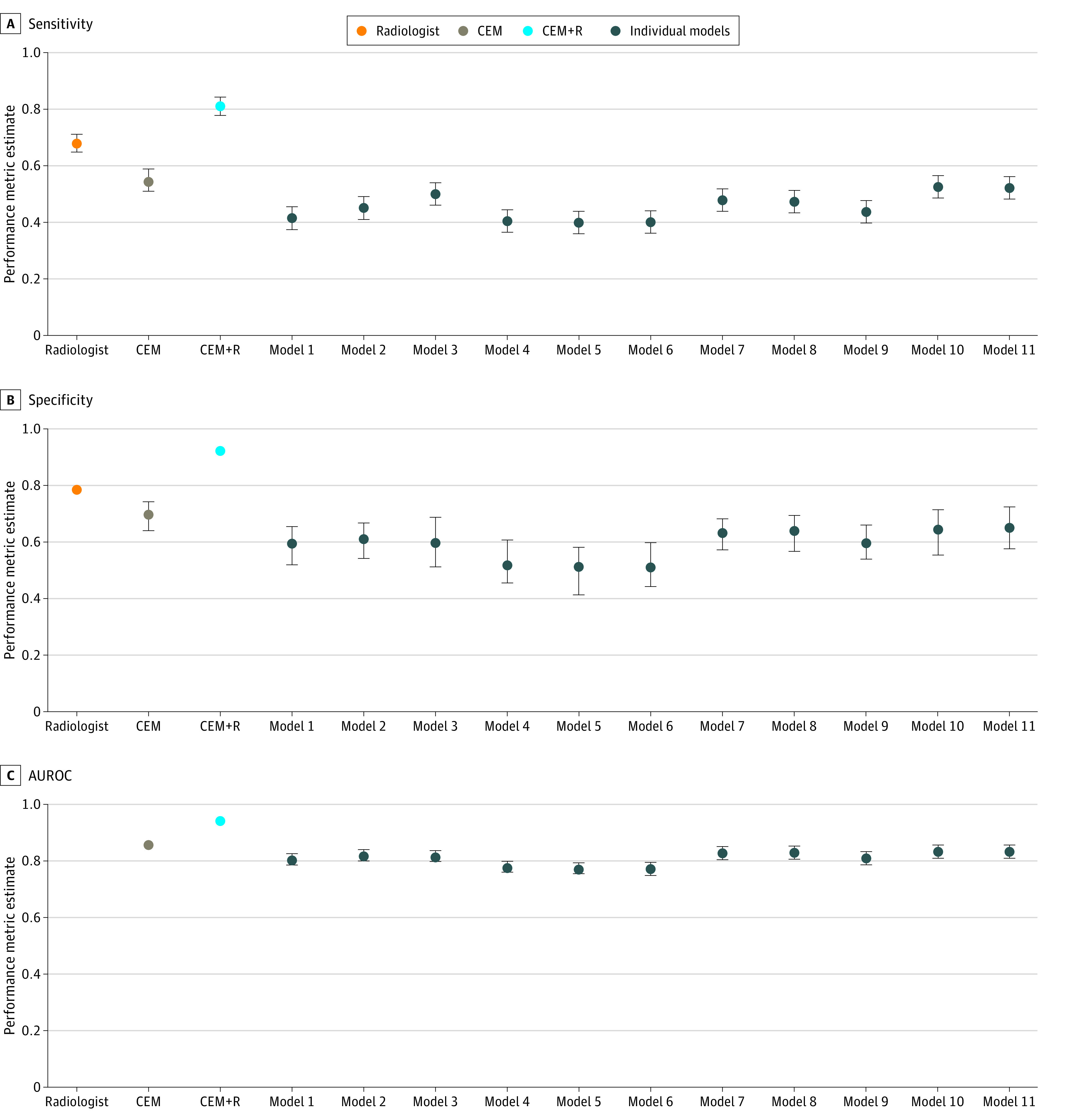
Comparison of Performance for Individual and Ensemble Models AUROC indicates area under the receiver operating characteristic curve; CEM, challenge ensemble method; CEM+R, challenge ensemble method plus radiologist; error bars, 95% CIs.

Across 11 individual models, the AUROC estimates ranged from 0.77 (95% CI, 0.75-0.79) to 0.83 (95% CI, 0.81-0.85). When evaluated at cut points selected to match radiologist sensitivity (0.826), the specificity of each individual model ranged from 0.509 (95% CI, 0.440-0.599) to 0.651 (95% CI, 0.572-0.723), lower than radiologist specificity (0.930; all *P* < .001). At the operating point matching radiologist specificity (0.930), models attained a range of 0.401 (95% CI, 0.360-0.440) to 0.527 (95% CI, 0.488-0.567) for sensitivity, lower than radiologist sensitivity (0.826; all *P* < .001). Figure 1 plots individual model ROC curves and points representing each cohort’s TP and FP rate of radiologist readers. Table 2 and Figure 2 summarize estimates for sensitivity, specificity, AUROC, including 95% CIs. eFigure 5 in the Supplement provides histograms for individual, CEM, and CEM+R models.

**Table 2.** Primary Performance Metrics of Individual and Ensemble Models

Model	Estimate (95% CI)		
	Sensitivity at radiologist specificity	Specificity at radiologist sensitivity	AUROC
Radiologist	0.826 (0.795-0.856)	0.930 (0.929-0.932)	NA
CEM	0.547 (0.508-0.588)	0.697 (0.637-0.749)	0.852 (0.836-0.869)
CEM+R	0.813 (0.781-0.843)	0.925 (0.916-0.934)	0.935 (0.923-0.945)
Individual model			
1	0.418 (0.374-0.460)	0.592 (0.516-0.652)	0.801 (0.781-0.820)
2	0.450 (0.410-0.491)	0.610 (0.539-0.669)	0.815 (0.798-0.833)
3	0.501 (0.461-0.542)	0.595 (0.506-0.689)	0.811 (0.791-0.831)
4	0.405 (0.365-0.447)	0.519 (0.454-0.610)	0.774 (0.754-0.794)
5	0.401 (0.360-0.440)	0.511 (0.413-0.583)	0.769 (0.748-0.789)
6	0.402 (0.362-0.443)	0.509 (0.440-0.599)	0.768 (0.747-0.788)
7	0.478 (0.438-0.523)	0.633 (0.571-0.684)	0.826 (0.809-0.844)
8	0.473 (0.433-0.515)	0.638 (0.565-0.698)	0.826 (0.807-0.844)
9	0.435 (0.393-0.478)	0.595 (0.538-0.659)	0.806 (0.787-0.825)
10	0.527 (0.488-0.567)	0.642 (0.550-0.715)	0.826 (0.806-0.847)
11	0.524 (0.481-0.565)	0.651 (0.572-0.723)	0.828 (0.809-0.849)

Abbreviations: AUROC, area under the receiver operating characteristic curve; CEM, challenge ensemble method; CEM+R, challenge ensemble method with radiologist; NA, not applicable.

### CEM Model Performance

The CEM model achieved an AUROC of 0.85 (95% CI, 0.84-0.87) in the UCLA cohort, lower than the performance achieved in the KPW (AUROC, 0.90) and KI (AUROC, 0.92) cohorts (Figure 1, Figure 2, Table 2). The CEM model also achieved lower sensitivity (0.547 [95% CI, 0.508-0.588] vs 0.826; *P* < .001) and specificity (0.697 [95% CI, 0.637-0.749] vs 0.930, *P* < .001) compared with radiologist outcomes. Similarly, secondary performance metrics of the CEM model were lower than those of the radiologist (eTable 6 in the Supplement). Calibration curves for CEM and CEM+R models are shown in eFigure 6 in the Supplement. Overall, both models had a relatively linear association with cancer risk, although they overestimated risk across the range of predicted risk.

### CEM+R Model Performance

Adding radiologist assessment to the CEM achieved a higher AUROC of 0.93 (95% CI, 0.92-0.95) compared with the CEM model without radiologist assessment (difference in AUROC, 0.08 [95% CI, 0.07-0.10]; *P* < .001). This performance was similar to CEM+R performance in KPW (AUROC, 0.94) and KI cohorts (AUROC, 0.94). We note that with the addition of a radiologist impression, the CEM+R model achieved a sensitivity (0.813 [95% CI, 0.781-0.843] vs 0.826; *P* = .20) and specificity (0.925 [95% CI, 0.916-0.934] vs 0.930; *P* = .18) similar to those of the radiologist. Secondary performance metrics of the CEM model were also similar to those of the radiologist (eTable 6 in the Supplement).

### Model Performance in by Subgroup of the UCLA Cohort

**Figure 3.**
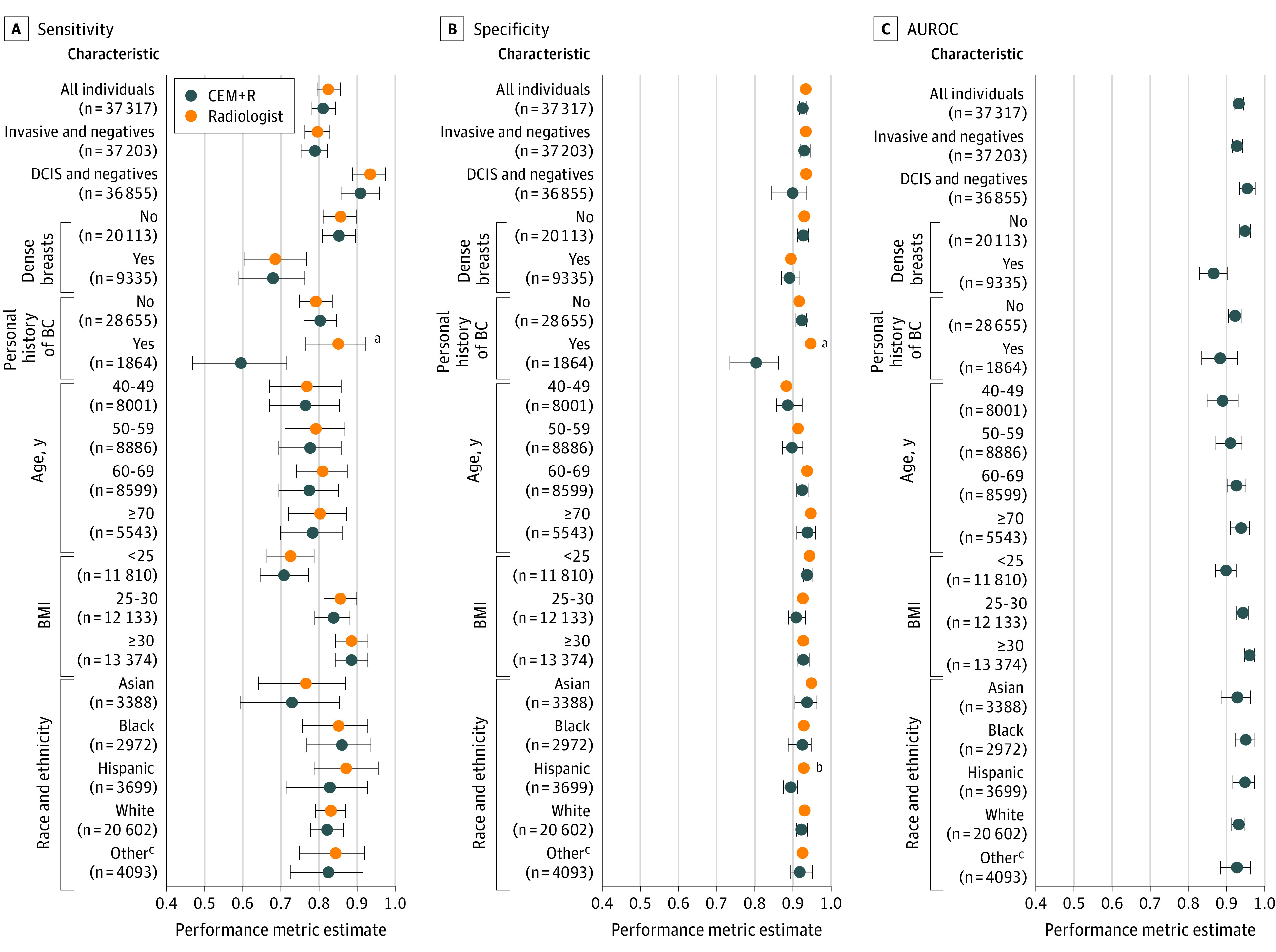
Performance of Radiologist and Challenge Ensemble Method With Radiologist (CEM+R) Models by Clinical Characteristic AUROC indicates area under the receiver operating characteristic curve; BC, breast cancer; BMI, body mass index (calculated as weight in kilograms divided by height in meters squared); DCIS, ductal carcinoma in situ; error bars, 95% CIs. ^a^Difference between radiologist and CEM+R with *P* < .001. ^b^Difference between radiologist and CEM+R with *P* = .004. ^c^Other included American Indian or Alaska Native, Native Hawaiian or other Pacific Islander, multiple races, or some other race.

As part of an exploratory analysis, we compared the CEM+R model performance with that of the original radiologist reader in 6 subgroups of the UCLA cohort, evaluating by cancer diagnosis, breast density, personal history of breast cancer, age, body mass index (calculated as weight in kilograms divided by height in meters squared), and race and ethnicity. Figure 3 illustrates trends within these subgroups. The CEM+R model and radiologist had significantly decreased performance for dense breasts compared with nondense breasts, particularly in terms of sensitivity (CEM+R: 0.680 [95% CI, 0.589-0.765] vs 0.853 [95% CI, 0.808-0.895]; *P* < .001; radiologist: 0.685 [95% CI, 0.602-0.768] vs 0.857 [95% CI, 0.811-0.900]; *P* < .001) and AUROC (CEM: 0.87 [95% CI, 0.83-0.90] vs 0.95 [95% CI, 0.93-0.96]; *P* < .001). With some notable exceptions, differences in performance between CEM+R and the radiologist within these subgroups were not statistically significant. The CEM+R model had significantly lower sensitivity (0.596 [95% CI, 0.466-0.717] vs 0.850 [95% CI, 0.466-0.717]; *P* < .001) and specificity (0.803 [95% CI, 0.734-0.861] vs 0.945 [95% CI, 0.936-0.954]; *P* < .001) than the radiologist in women with a prior history of breast cancer (1864 of 30 519 examinations with history of breast cancer data). CEM+R specificity was also lower in Hispanic women (0.894 [95% CI, 0.873-0.910] vs 0.926 [95% CI, 0.919-0.933]; *P* = .004). CEM+R sensitivity was higher for DCIS than for invasive cancers. Sensitivity, specificity, and AUROC were worse for women with dense breasts vs those with nondense breasts. The calibration intercept and slope for the CEM+R model within each subgroup are shown in eFigure 6 in the Supplement. Relative performance patterns in subgroups were similar to those found for AUROC in Figure 3.

## Discussion

In this diagnostic study, we examined the performance of CEM and CEM+R models developed as part of the DREAM Mammography Challenge^9^ in an independent, diverse patient population from the UCLA health system. These models were developed and tested using a large cohort from western Washington state and externally validated using a Swedish cohort, both predominantly representing White screening populations. Despite being trained and externally validated on 2 large data sets, individual models fared poorly when applied to the UCLA cohort. Using performance thresholds to match the mean UCLA radiologist performance (0.826 sensitivity or 0.930 specificity), individual models performed significantly worse, with a sensitivity ranging from 0.401 to 0.527 and specificity ranging from 0.509 to 0.651. These results suggest that the clinical adoption of any individual model without further refinement (eg, fine-tuning model parameters) would not be recommended. CEM performed better than individual AI models, with a sensitivity of 0.547 and a specificity of 0.697, which was still significantly worse than the radiologist performance. Combining the impression of the radiologist and CEM, the CEM+R model achieved similar performance to the radiologist, with a sensitivity of 0.813 and a specificity of 0.925.

A 2022 systematic review^15^ examined 13 studies that included external validation components in their analyses of AI algorithms for automated mammography interpretation. A 2020 study^16^ externally validated 3 commercially available breast-screening AI algorithms, finding that 1 of 3 algorithms performed significantly better than the others and outperformed human readers in a European cohort. The study, however, revealed few details about each algorithm or the cohorts on which they were trained. It is unclear whether the algorithms’ performance will generalize, particularly whether the algorithm that performed better than human readers will generalize to other cohorts. Schaffter et al^9^ found that combining multiple models yielded the highest diagnostic accuracy, a result that was consistent with our study’s findings. In our study, we used cancer outcome information from an institutional registry, including cancer diagnoses for women who may have received biopsies or diagnoses outside of UCLA. These numbers reflect reader performance in clinical practice more than audit reports generated as a requirement of the Mammography Quality Standards Act. Of note, 2 other studies identified in the systematic review^15^ performed this step. Our study found that several factors were associated with AI performance for mammography interpretation. Model and radiologist performance were lower in women with dense breasts. Dense breast tissue reduces the visibility of masses by affecting the contrast between fat and tissue, complicating the ability of radiologists (and AI and ML algorithms) to detect abnormalities. Another notable trend is poor performance in women with a personal history of breast cancer. Most DREAM Challenge models were developed using publicly available data sets, usually without examinations from women with a personal history of breast cancer. Lumpectomy scars among these women mimic cancers on mammography, making the evaluation of postlumpectomy mammograms more difficult. Finally, the sensitivity of DCIS lesions, usually represented by calcifications rather than masses, was higher than that of invasive cancer lesions for radiologists and AI models.

While the CEM+R model achieved similar AUROCs across racial and ethnic groups, it should be noted that model specificity was significantly lower among Hispanic women compared with the radiologist. The CEM+R model and radiologist had lower sensitivity in Asian women compared with women of other races. We note that the original distribution of KPW; public data sets, such as the Curated Breast Imaging Subset of the Digital Database for Screening Mammography, commonly used to train models; and the external validation KI cohort consisted of examinations predominantly from White women. Our results support the need for increased diversity in training data sets, particularly for women in minority racial and ethnic groups, women with dense breasts, and women who have previously undergone surgical resection. Our results also reinforce the importance of transparency regarding model training, including cohort selection bias, by reporting detailed inclusion criteria and providing distributions around demographic variables, clinical factors associated with risk, and cancer outcomes. Imaging-based AI should also report what protocols and imaging equipment are used. PROBAST is a risk of bias assessment tool that may also serve as guidance for reporting.^17^

### Limitations

Several limitations of this study are noted. We used a subset of patients weighted to match the full cohort. Patients had to be excluded from the analysis due to technical issues (eg, inability to retrieve imaging examinations from PACS) and missing clinical data that were required to execute the models, a potential source of selection bias. As with the original DREAM Challenge, the mammography images were weakly labeled, having only examination-level determination of whether cancer was diagnosed within 12 months. Cancers were not localized on the mammography images. Given that only Hologic equipment was used at UCLA, the algorithm was not evaluated against images acquired using other vendor equipment. Cancer outcome information was obtained from a regional registry, which did not capture outcomes for patients who may have been diagnosed outside of the region and so could have been incorrectly identified as TNs. In addition to examining the accuracy and reliability of the model, the explainability and fairness of the model’s outputs, core aspects of model trustworthiness,^18^ were not fully explored in this study.

## Conclusions

This diagnostic study examined the performance of CEM and CEM+R models in a large, diverse population that had not been previously used to train or validate AI or ML models. The observed performance suggested that promising AI models, even when trained on large data sets, may not necessarily be generalizable to new populations. Our study underscores the need for external validation of AI models in target populations, especially as multiple commercial algorithms arrive on the market. These results suggest that local model performance should be carefully examined before adopting AI clinically.

## Notes

Supplement. **eAppendix 1.** Study Population **eAppendix 2.** Case Selection and Weighting **eAppendix 3.** Challenge Ensemble Method **eAppendix 4.** Model Execution **eAppendix 5.** Analysis of Model Outputs **eAppendix 6.** Code Availability **eTable 1.** Clinical Variables With Values and Sources **eTable 2.** Missingness of Clinical Risk Factors **eTable 3.** *International Classification of Diseases for Oncology, Third Revision* (*ICD-O*) Codes Identified as Breast Cancer **eTable 4.** Inverse Probability Weights Used to Make Analyzed Subset Representative of Original Cohort **eTable 5.** Comparison Between Training and Evaluation Cohorts **eTable 6.** Comparison of Model and Radiologist Performance **eFigure 1.** Cohort Selection **eFigure 2.** Breakdown of Analyzed Subset Selected and Successfully Retrieved for Analysis **eFigure 3.** Target Metrics After Inverse Probability Weighting **eFigure 4.** Architecture of Evaluation Environment **eFigure 5.** Histograms of Model Predictions **eFigure 6.** Calibration Performance of Challenge Ensemble Method With Radiologist Models By Clinical Characteristic
eReferences
