# Supplementary material for: External Validation of an Ensemble Model for Automated Mammography Interpretation by Artificial Intelligence
Source: JAMA Netw Open. 2022 Nov 21;5(11):e2242343. doi: 10.1001/jamanetworkopen.2022.42343 (PMC9679879; doi:10.1001/jamanetworkopen.2022.42343)
Supplement: Supplement. — eAppendix 1. Study Population eAppendix 2. Case Selection and Weighting eAppendix 3. Challenge Ensemble Method eAppendix 4. Model Execution eAppendix 5. Analysis of Model Outputs eAppendix 6. Code Availability eTable 1. Clinical Variables With Values and Sources eTable 2. Missingness of Clinical Risk Factors eTable 3. International Classification of Diseases for Oncology, Third Revision (ICD-O) Codes Identified as Breast Cancer eTable 4. Inverse Probability Weights Used to Make Analyzed Subset Representative of Original Cohort eTable 5. Comparison Between Training and Evaluation Cohorts eTable 6. Comparison of Model and Radiologist Performance eFigure 1. Cohort Selection eFigure 2. Breakdown of Analyzed Subset Selected and Successfully Retrieved for Analysis eFigure 3. Target Metrics After Inverse Probability Weighting eFigure 4. Architecture of Evaluation Environment eFigure 5. Histograms of Model Predictions eFigure 6. Calibration Performance of Challenge Ensemble Method With Radiologist Models By Clinical Characteristic eReferences [file jamanetwopen-e2242343-s001.pdf]

Supplemental Online Content

Hsu W, Hippe DS, Nakhaei N, et al. External validation of an ensemble model for automated mammography interpretation by artificial intelligence. *JAMA Netw Open.* 2022;5(11):e2242343. doi:10.1001/jamanetworkopen.2022.42343

eAppendix 1. Study Population
eAppendix 2. Case Selection and Weighting
eAppendix 3. Challenge Ensemble Method
eAppendix 4. Model Execution
eAppendix 5. Analysis of Model Outputs
eAppendix 6. Code Availability
eTable 1. Clinical Variables With Values and Sources
eTable 2. Missingness of Clinical Risk Factors
eTable 3. *International Classification of Diseases for Oncology, Third Revision* (*ICD-O*) Codes Identified as Breast Cancer
eTable 4. Inverse Probability Weights Used to Make Analyzed Subset Representative of Original Cohort
eTable 5. Comparison Between Training and Evaluation Cohorts
eTable 6. Comparison of Model and Radiologist Performance
eFigure 1. Cohort Selection
eFigure 2. Breakdown of Analyzed Subset Selected and Successfully Retrieved for Analysis
eFigure 3. Target Metrics After Inverse Probability Weighting
eFigure 4. Architecture of Evaluation Environment
eFigure 5. Histograms of Model Predictions
eFigure 6. Calibration Performance of Challenge Ensemble Method With Radiologist Models By Clinical Characteristic
eReferences

This supplemental material has been provided by the authors to give readers additional information about their work.

© 2022 Hsu W et al. *JAMA Network Open.*

eAppendix 1. Study Population

Our analysis was conducted under an institutional review board-approved protocol (IRB#19-000849). We utilized clinical, imaging, and cancer outcomes data collected as part of the Athena Breast Health Network (referred to as Athena), an observational study conducted across breast screening programs at five University of California medical centers, including UCLA Health.[1] The goal of Athena was to advance understanding of breast cancer risk and the trajectory of different breast cancer subtypes, supporting the development of improved risk models. Data for this study come from the UCLA Athena data. At UCLA, breast screening is performed at ten geographically distributed imaging centers that acquire screening and diagnostic mammography images using equipment primarily from one vendor, Hologic (Marlborough, MA, USA). Women who arrived at an outpatient imaging center for a mammographic or ultrasound breast imaging exam (screening or diagnostic) completed an electronic or hard copy survey related to their health history, lifestyle behaviors, and family history of cancer. Study accrual spanned December 2010-October 2015. The UCLA Athena cohort enrolled 49,244 women who completed 89,881 surveys during the study accrual period. A list of breast imaging exams for these women was obtained for the study accrual period plus an additional four years, spanning December 2010-December 2019. This analysis focused specifically on 2D screening mammography to match what was used in the original DREAM Mammography challenge. **eFigure 1** summarizes the patient selection process.

Clinical variables. During the training phase of the DREAM Challenge,[2] participants were given access to 14 clinical/demographic variables and the mammography images for each case. **eTable 1** enumerates the clinical variables and how they were recorded as part of the Challenge. During the accrual period of the Athena study, women were asked to answer a survey around the time of their breast imaging visit, with each woman completing at least one survey. From the survey, we obtained information on variables such as self-reported race/ethnicity, personal and family history of breast cancer, use of hormone replacement therapy, and whether the individual has breast implants. We categorized survey responses to be consistent with the DREAM Challenge (**eTable1)**. **eTable 2** summarizes the percentage of data available for each covariate collected in the survey. For imaging obtained after the accrual period (November 2015-December 2019), patients were no longer asked to complete a survey. In this situation, we imputed information about the clinical variables from observations recorded in the electronic health record (e.g., documented breast cancer history before screening exam, a record of having undergone a breast implant screening exam protocol). The woman's race/ethnicity was assumed not to change and used as the last known value. Variables that were not imputable were left missing. Body mass index (BMI) is the only variable required to be specified for the model to execute.

Given that height and weight were not collected at the screening exam, we imputed these values from the flowchart in the electronic health record. Our approach for imputation is as follows: 1) for height, we used any measurement that was available in the woman's record; and 2) for weight, we used any available weight measurement within one year from the time of the imaging exam, which was consistent with the DREAM Challenge. We picked the closest measurement to the exam date with a preference for pre-exam measurements. If more than one measurement was performed on a given date, we selected the most recent measurement obtained. Overall, BMI values for 31,204 exams (25.6%) were imputed using this approach.

Screening exams. We searched our radiology information system for mammography screening exams associated with the 49,244 women in the UCLA Athena population between December 1, 2010 and December 31, 2019, resulting in 184,935 exams being identified. Excluding ultrasound (n=8,517) and digital breast tomosynthesis (n=54,665) exams, excluding 7,901 women who only had non-2D screening exams, the complete 2D UCLA Athena cohort had 41,343 women and 121,753 exams from which cases were selected for inclusion, see **eAppendix 2**. Among the 41,343 women in the 2D UCLA Athena cohort, 10,562 (25.5%) had a single screening exam, 9,424 (22.8%) had two exams, 7,662 (18.5%) had three exams, and 13,695 (33.1%) had four or more exams. Exams were originally interpreted by one of fifteen fellowship-trained radiologists. All exams were processed using a computer-aided detection algorithm, Hologic R2, prior to review by the radiologist.

Cancer outcomes. Our institutional cancer registry maintains a database of cancer cases abstracted using information from hospitals within the Southern California region. We obtained an extract from the cancer registry in January 2021. Information about diagnosed cancers was coded using ICD-O-3. Among the 41,343 women considered, 723 had at least one diagnosis of breast cancer (DCIS and invasive). Given some variability in how the cancer cases were coded (e.g., some multiple histology diagnoses were associated with an ICD-O-3 code), we reviewed all the diagnoses and

curated a set of ICD-O-3 codes related to breast cancer according to the guideline on the North American Association of Central Cancer Registries (NAACCR) website (**eTable 3)**.

© 2022 Hsu W et al. *JAMA Network Open.*

eAppendix 2. Case Selection and Weighting

We identified a subset of exams for our analysis using the available clinical, imaging, and cancer outcomes data. Exams were partitioned into four groups: true positives (TP, screening exam marked as BI-RADS 0/3/4/5 and had a cancer diagnosis within 12 months), false negatives (FN, screening exam read as BI-RADS 1/2 and had a cancer diagnosis within 12 months), false positive (FP, screening exam read as BI-RADS 0/3/4/5 but no cancer diagnosis within 12 months), and true negatives (TN, consecutive exams read as BI-RADS 1/2 at least 12 months apart without a cancer diagnosis noted within this period). Of the 121,753 2D exams within the 2D UCLA Health Athena cohort, 723 (0.6%) exams had a cancer diagnosis within 12 months. The original radiologist interpretation yielded 597 (0.5%) true positives, 126 (0.1%) false negatives, 8,432 (6.9%) false positives, and 112,598 (92.5%) were true negatives.

Our initial target was to retrieve all exams associated with cancer diagnoses (n=723) and a subsample of negative cases. Of all the exams related to cancer, 147 exams were excluded for the following reasons: 1) the exam could not be downloaded from the PACS (N=12); the exam did not have a standard set of four screening images or was mislabeled as 2D (N=20); or the exam had missing BMI values (n=97).

Due to differences in TP, FN, FP, and TN distributions between the 2D UCLA Health Athena cohort and the analyzed subset, performance metrics in the validation cohort were computed using inverse probability of selection weighting. The following proportions (number of exams in the analyzed subset divided by the number of exams in the entire 2D UCLA Health Athena cohort) of TP, FN, FP, and TN were included in the analysis: 0.779 (465/597), 0.881 (111/126), 0.412 (3,474/8,432), and 0.295 (33,267/112,598), respectively. The inverse probability weights were calculated as the inverse of these proportions (**eTable 4).** Estimates of performance metrics were weighted using these weights so the corresponding parameter estimates would reflect the proportions of these groups (TP, FN, FP, and TN) in the full 2D UCLA Health Athena cohort. The number of false positives and true negatives were selected to achieve an unweighted abnormal interpretation (recall) rate of 9-10%, which was consistent with the rate observed in the 2D UCLA Health Athena cohort during the study period. **eFigure 2** and **eTable 4** summarize the analyzed subset of successfully retrieved exams that were run through the models. **eFigure 3** illustrates the impact of reweighting on calculated measures of radiologist performance. After applying the weights, the apparent performance of the radiologist in the analyzed subset matched the radiologist's performance when estimated from the full 2D UCLA Health Athena cohort. **eTable 5** compares basic characteristics of the training (KPW) and evaluation cohorts (Karolinska Institute and UCLA).

© 2022 Hsu W et al. *JAMA Network Open.*

eAppendix 3. Challenge Ensemble Method

The Challenge Ensemble Method (CEM) comprises eleven models contributed by the top six performing competitive phase teams in the DREAM Mammography Challenge. Models were distributed as Docker containers and were retrieved from a Docker repository hosted in Synapse. Each model was treated as a "black box" in that no modifications were made to the algorithms before running them on our dataset. All models followed a consistent specification regarding how input variables are defined and how the model outputs are formatted. Each model expects a list of exams and a predefined set of metadata, and a directory of the images as inputs. Once executed, each model generated a set of standardized outputs, including a confidence score between 0 and 1, reflecting the likelihood of cancer for each side of the breast.

The CEM used confidence score outputs from each model as inputs, reweighting them and outputting a combined score.[1] The CEM with radiologist suspicion (CEM+R) is the ensemble model with the added input of the overall BI-RADS score provided by the original interpreting radiologist at the exam level. The BI-RADS score was binarized into low (BI-RADS 1/2) and high (BI-RADS 0/3/4/5) suspicion, then added as an additional independent variable. The model weights, including the radiologist suspicion, were trained using the original training (KPW) dataset and were not altered for this analysis.

© 2022 Hsu W et al. *JAMA Network Open.*

eAppendix 4. Model Execution

Given the number of models and images that would be executed, we established a distributed evaluation environment within Amazon Web Services, illustrated in **eFigure 4**, to parallelize the execution and provide each model with sufficient resources. Each submitted model had exclusive access to 1 Tesla K80 GPU, 22 CPU cores, 200GB memory, and 200GB of scratch space in the original Challenge. In our implementation, execution of the 11 models was distributed across three EC2 instances: one p2.8xlarge instance (32 CPU cores, a Tesla K80 GPU, and 61GB memory) and two g3.8xlarge (32 CPU cores, two Tesla M60 GPUs, 244GB memory). Each instance had access to 1TB scratch space. A script was written to automate the management and execution of each exam across the eleven models. The script queries the clinical and imaging archive to generate two files: 1) *exam metadata,* which contains clinical/demographic information for each exam such as exam identifier, BMI, etc; and 2) *image crosswalk*, which links individual images to their respective exams, including laterality (right or left) and view (CC or ML). Exams were executed in batches of 2,000 to ensure sufficient computational resources for each model. Each exam took 6 minutes to complete all individual models and the ensemble on average. For each batch of exams, a model outputs a file that contains the exam identifier, laterality, and probability of cancer.

eAppendix 5. Analysis of Model Outputs

Once all model executions were completed, a script was developed to concatenate results from all batches and models, resulting in a single file that contains the confidence scores for all models across each exam and laterality. Individual model predictions were generated on the breast-level. They were aggregated to the exam-level using the maximum probability of cancer computed between the left and right sides as output for that model and exam. **eFigure 5** provides a histogram of the outputted probabilities for each model. The CEM then took each of the individual model outputs as inputs to compute a weighted combination to derive an estimate of the probability of cancer, where the weights were originally derived from the DREAM Mammography Challenge training set. In addition to each model, the CEM+R incorporated the radiologist's assessment as a binary variable where BI-RADS 0, 3, 4, and 5 were set to 1, and BI-RADS 1 and 2 were set to 0. Radiologist and model performance was assessed at the exam-level, consistent with the DREAM Mammography Challenge. The calibration intercept and slope for the CEM+R model across subgroups are shown in **eFigure 6**.

© 2022 Hsu W et al. *JAMA Network Open.*

eAppendix 6. Code Availability

We have shared the code and scripts for running the Challenge Ensemble Method at https://github.com/hsu-lab/DigitalMammographyEnsembleValidation.

The information on the original ensemble execution environment and models can be found at Sage-Bionetwork on GitHub[2]. The site has scripts and documents for running ensemble models using digital mammography images encoded in DICOM format.

The DREAM Mammography Challenge consisted of two parts: Sub Challenge 1 (SC1) and Sub Challenge 2(SC2). The details of SC1 and SC2 are described on the Digital Mammography DREAM Challenge website[3]. This website also contains links for downloading the Docker image of each model. If one cannot download the Docker images, s/he needs to contact Dream Challenge organizers for the permissions to gain access to the images.

For the external validation study, we used each model's SC2 implementation. Eleven models were used in the original ensemble paper[1]. The identifiers of the eleven models are found in the file ensemble_job.cwl. The 'cwl' stands for Common Workflow Language, which the DREAM Challenge organizers used to queue the launch of the eleven models. Instead of dealing with the overhead of executing the CWL script, we developed a simplified script to launch each of the eleven Docker images. This simplified approach allowed us to:

- Manually execute Docker images using sample data to verify the successful execution of each model.
- Re-launch Docker images in the situation when the model execution fails. The model execution could be restarted from a breakpoint without needing to re-run the entire experiment.
- Divide the entire test set into sub-collections.

Through empirical experimentation running the models, we allocated each model to one of the three EC2 instances.

All models in the ensemble were given the same data as input and in a standardized format, as follows:

- One directory containing an exam metadata file and an imaging crosswalk file
- One directory containing the images in DICOM format

Only screening exams with all four views ['R CC', 'R MLO', 'L CC', 'L MLO'] were used; the series description field in the DICOM header was used to confirm the presence of all four views. A qualified imaging exam combined with available exam metadata (**eTable 1)**, resulting in one entry in the metadata file, four entries in the imaging crosswalk file, and four DICOM files for each of the required views are copied into the imaging directory. Each imaging exam was uniquely identified by a patient ID and imaging exam ID in the metadata and image crosswalk files.

© 2022 Hsu W et al. *JAMA Network Open.*

eTable 1. Clinical Variables With Values and Sources

Description	Permissible Values	Source
Unique identifier assigned to each woman	Integer	Auto assigned
Unique identifier assigned to each imaging exam	Integer	Auto assigned
Imaging exam procedure description	Free-text	Radiology information system
Imaging exam procedure code	Free-text	Radiology information system
Days from time of imaging exam to time survey was completed	Integer (+/- days from exam)	Calculated
Overall BI-RADS score	0=need additional imaging evaluation<br>1=negative<br>2=benign findings<br>3=probably benign<br>4(A/B/C)=suspicious abnormality<br>5=highly suspicious of malignancy<br>6=known biopsy with proven malignancy	Radiology information system
BI-RADS breast density type	A=almost entirely fatty<br>B=scattered areas of dense fibroglandular breast tissue<br>C=areas of glandular and connective tissue<br>D=extremely dense	Extracted from narrative using natural language processing
Age at the time of survey completion	Integer	Calculated
Ever had implants (survey response)	0=Never<br>1=Ever had implants	Survey
Laterality of the implant(s) in place today (survey response)	1=Right breast only<br>2=Left breast only<br>4=Bilateral<br>5=Yes, woman-level info only	Survey
Personal history of breast cancer (survey response)	0=None<br>1=Yes	Survey
Years since prior breast cancer diagnosis	Integer	Survey
Laterality of prior breast cancer	1=Right breast only<br>2=Left breast only<br>3=Unspecified laterality<br>4=Bilateral	Survey
History of breast reduction	0=None<br>1=Prior breast reduction	Survey
Laterality of breast reduction	1=Right breast only<br>2=Left breast only<br>4=Bilateral	Survey

© 2022 Hsu W et al. *JAMA Network Open.*

Current use of hormone replacement therapy	0=No<br>1=Yes<br>9=Unknown	Survey
Current use of anti-estrogen therapy (Tamoxifen/Raloxifene)	0=No<br>1=Yes<br>9=Unknown	Survey
First degree relative with BC	0=No<br>1=Yes<br>9=Unknown	Survey
First degree relative with BC < 60 years old	0=No<br>1=Yes<br>9=Unknown	Survey
BMI at screening exam	Decimal	Flowsheet
Race and ethnicity	1=White<br>2=Black<br>3=Asian<br>4=Hawaiian/Pacific Islander<br>5=American Indian/Alaska Native<br>6=Other<br>7=Mixed<br>8=Hispanic<br>9=Missing	Survey
Age at time of pathology-confirmed diagnosis	Integer	Cancer registry
Text description of pathology diagnosis	Text	Cancer registry
ICD-O-3 SEER site code	Alphanumeric code	Cancer registry
Histology behavior code (ICD-O-3)	Alphanumeric code	Cancer registry
Breast cancer diagnosis flag	INV=Invasive cancer<br>DCIS=Ductal carcinoma in situ	Cancer registry
Breast laterality of cancer diagnosis	LEFT(2)=Left<br>RIGHT(1)=Right<br>BILAT SIMULT(4)=Bilateral	Cancer registry
Days from imaging exam to cancer diagnosis	Integer	Calculated

BI-RADS: Breast Imaging Reporting and Data System; BC: breast cancer; BMI: body mass index; SEER: Surveillance, Epidemiology, and End Results program

© 2022 Hsu W et al. *JAMA Network Open.*

eTable 2. Missingness of Clinical Risk Factors

Description	% Available data
Overall BI-RADS score	100%
BI-RADS breast density type	51.3%
Age at the time of survey completion	51.7%
Ever had implants (survey response)	48.3%
Laterality of the implant(s) in place today (if response to "Ever had implants" was "Yes")	100%
Personal history of breast cancer (survey response)	50.3%
Years since prior breast cancer diagnosis (if "Prior history of breast cancer" was "Yes")	7.2%
Laterality of prior breast cancer (if "Prior history of breast cancer" was "Yes")	19.0%
History of breast reduction	51.7%
Laterality of breast reduction (if "History of breast reduction" was "Yes")	100%
Current use of hormone replacement therapy	51.7%
Current use of anti-estrogen therapy (Tamoxifen/Raloxifene) (if "Current use of hormone replacement therapy" was "Yes")	100%
First degree relative with BC	51.7%
First degree relative with BC < 60 years old (if "First degree relative with BC" is "Yes")	29.6%
BMI*	40.6%
Race*	52.1%

BI-RADS: Breast Imaging Reporting and Data System; BC: breast cancer; BMI: body mass index
*Missingness of BMI and race prior to imputation.

© 2022 Hsu W et al. *JAMA Network Open.*

eTable 3. *International Classification of Diseases for Oncology, Third Revision* (*ICD-O*) Codes Identified as Breast Cancer

CATEGORY	HISTOLOGY BEHAVIOR CODE (ICD-O-3)		
Invasive	• 85003<br>• 85203<br>• 82113<br>• 85223<br>• 85243<br>• 85002<br>• 80103<br>• 84803<br>• 81403<br>• 85753	• 85233<br>• 82003<br>• 85033<br>• 85073<br>• 84013<br>• 85232<br>• 80503<br>• 82013<br>• 85433<br>• 80133	• 85103<br>• 84903<br>• 85303<br>• 85413<br>• 80003<br>• 88003<br>• 90203<br>• 88013<br>• 80323
DCIS	• 85222<br>• 82012<br>• 82302<br>• 85232	• 85012<br>• 85032<br>• 85072<br>• 85042	• 84012<br>• 82603

© 2022 Hsu W et al. *JAMA Network Open.*

eTable 4. Inverse Probability Weights Used to Make Analyzed Subset Representative of Original Cohort

Group	Original Cohort*	Analyzed Subset*	Proportion Analyzed†	Inverse Probability Weight‡
TN	112,598 (92%)	33,267 (89%)	0.295	3.38
FP	8,432 (6.9%)	3,474 (9.3%)	0.412	2.43
TP	597 (0.5%)	465 (1.2%)	0.779	1.28
FN	126 (0.1%)	111 (0.3%)	0.881	1.14
Total	121,753 (100%)	37,317 (100%)	0.306	

TN = true negative; FP = false positive; TP = true positive; FN = false negative;
*No. (column percentage);
†Proportion of each group in the original cohort that was included in the analysis subset;
‡Weight = 1 / (proportion analyzed).

© 2022 Hsu W et al. *JAMA Network Open.*

eTable 5. Comparison Between Training and Evaluation Cohorts

	KPW		KI	UCLA	
**Characteristic**	**Training**	**Evaluation**	**Evaluation**	**Evaluation (Full)**	**Evaluation (Analyzed)***
Screening exams, no	100,974	43,257	166,578	121,753	37,317
Women, no	59,923	25,657	68,008	41,343	26,817
Women diagnosed with BC within 12 mo of mammogram, N, %	669 (1.1%)	283 (1.1%)	780 (1.1%)	714 (1.7%)	573 (2.1%)
Invasive BCs	495 (74.0%)	202 (71.4%)	681 (87.3%)	567 (79.4%)	487 (85.0%)
DCIS	174 (26.0%)	81 (28.6%)	99 (12.7%)	147 (20.6%)	86 (15.0%)
Women without a BC diagnosis within 12 mo, N, %	59,254 (98.9%)	25,374 (98.9%)	67,228 (98.9%)	40,629 (98.3%)	26,244 (97.9%)
Age, mean (SD), y	58.4 (9.7 s.d.)	58.4 (9.7 s.d.)	53.3 (9.4 s.d.)	58.9 (11.6 s.d.)	58.4 (11.5 s.d.)
BMI, mean (SD)	28.2 (6.9 s.d.)	28.1 (6.8 s.d.)	NA	28.4 (6.9 s.d.)	29.0 (7.0 s.d.)

*In the analyzed subset of the UCLA cohort, cancers and radiologist false positives were oversampled to maintain large sample sizes; inverse probability of selection weights were used so that weighted averages would reflect values from the full UCLA cohort.
KPW: Kaiser Permanente Washington; KI: Karolinska Institute; UCLA: UCLA Athena Breast Health Network
s.d.: standard deviation

© 2022 Hsu W et al. *JAMA Network Open.*

eTable 6. Comparison of Model and Radiologist Performance

		At Radiologist Specificity								At Radiologist Sensitivity							
		CEM				CEM+R				CEM				CEM+R			
**Metric**	**Radiologist**	**Estimate**	**Δ***	**(95% CI)**	**P-value**	**Estimate**	**Δ***	**(95% CI)**	**P-value**	**Estimate**	**Δ***	**(95% CI)**	**P-value**	**Estimate**	**Δ***	**(95% CI)**	**P-value**
Sensitivity	0.826	0.547	-0.278	(-0.322, -0.233)	<0.001	0.813	-0.012	(-0.032, 0.007)	0.20								
Specificity	0.930									0.697	-0.234	(-0.295, -0.182)	<0.001	0.925	-0.006	(-0.014, 0.004)	0.18
PPV	0.066	0.045	-0.021	(-0.025, -0.017)	<0.001	0.065	-0.001	(-0.002, 0.000)	0.18	0.016	-0.050	(-0.056, -0.044)	<0.001	0.062	-0.005	(-0.011, 0.004)	0.19
NPV	0.999	0.997	-0.002	(-0.002, -0.001)	<0.001	0.999	0.000	(0.000, 0.000)	0.20	0.999	0.000	(-0.001, 0.000)	<0.001	0.999	0.000	(0.000, 0.000)	0.51
AIR	0.074	0.073	-0.002	(-0.002, -0.001)	<0.001	0.074	0.000	(0.000, 0.000)	0.42	0.306	0.232	(0.181, 0.293)	<0.001	0.080	0.006	(-0.004, 0.014)	0.18
CDR	0.005	0.003	-0.002	(-0.002, -0.001)	<0.001	0.005	0.000	(0.000, 0.000)	0.20	0.005	0.000	*	*	0.005	0.000	*	*
FNR	0.001	0.003	0.002	(0.001, 0.002)	<0.001	0.001	0.000	(0.000, 0.000)	0.20	0.001	0.000	*	*	0.001	0.000	*	*

ROC = receiver operating characteristic; AUC = area under the curve; CI = confidence interval; CEM = challenge ensemble method; CEM+R = challenge ensemble method + radiologist; PPV = positive predictive value; NPV = negative predictive value; AIR = abnormal interpretation rate; CDR = cancer detection rate; FNR = false negative rate ([false negatives] / N); Δ = difference in performance relative to radiologist (ensemble - radiologist);

*The difference between the ensemble models and the radiologist for CDR and FNR is forced to be zero in this case because the model prediction cutpoint was selected to match radiologist sensitivity, making TP and FN the same for both.

© 2022 Hsu W et al. *JAMA Network Open.*

eFigure 1. Cohort Selection

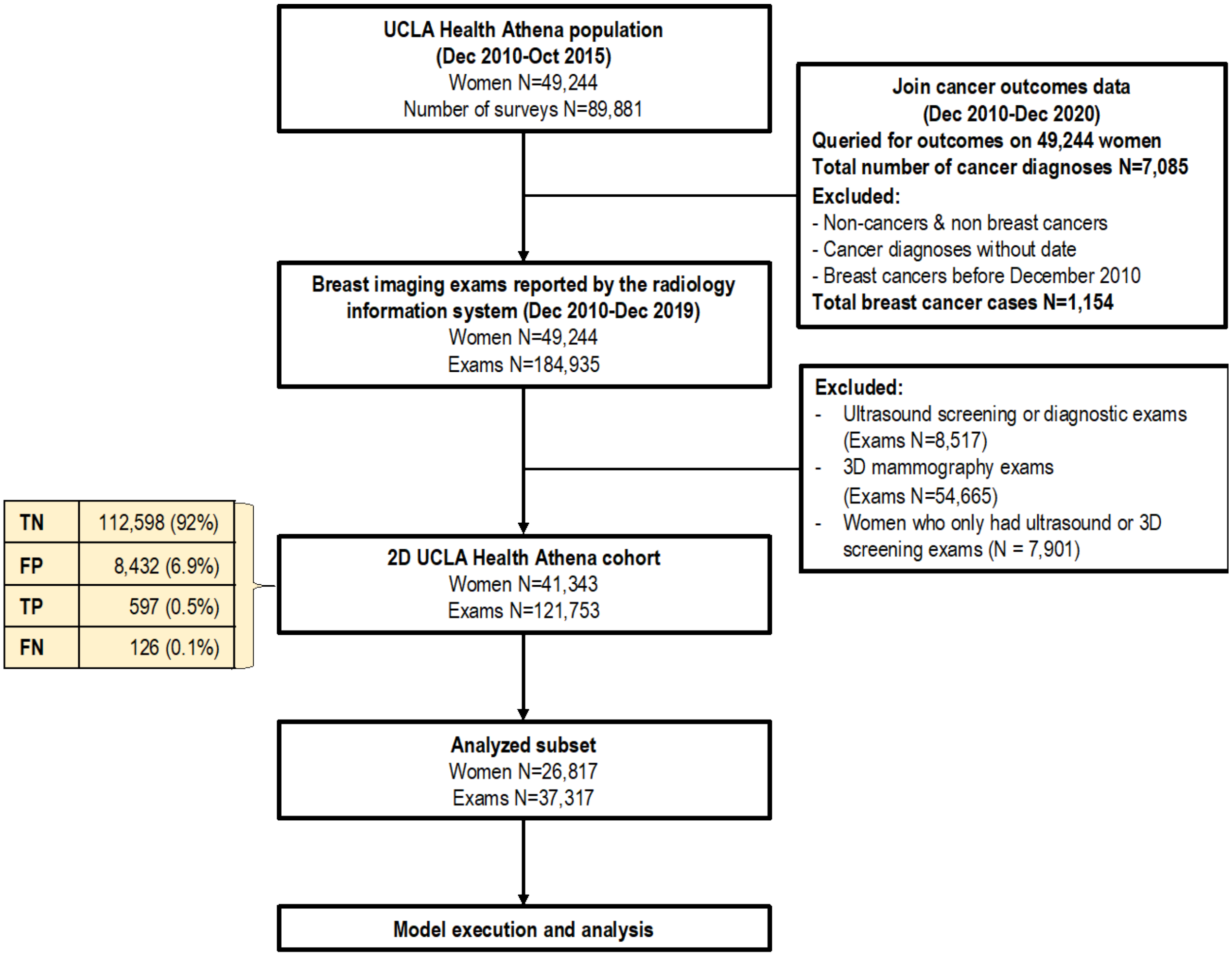

© 2022 Hsu W et al. *JAMA Network Open.*

eFigure 2. Breakdown of Analyzed Subset Selected and Successfully Retrieved for Analysis

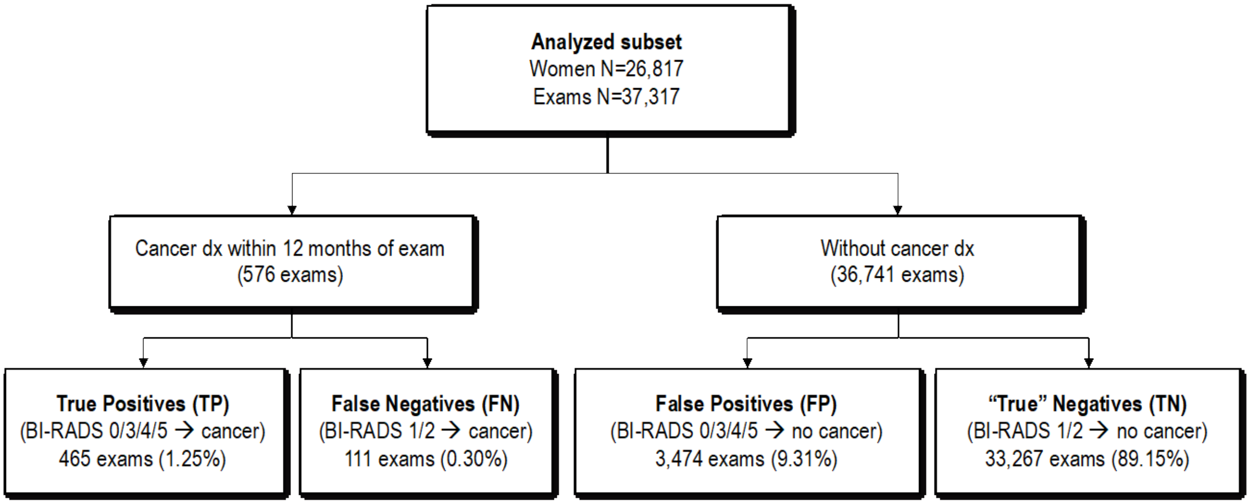

© 2022 Hsu W et al. *JAMA Network Open.*

eFigure 3. Target Metrics After Inverse Probability Weighting

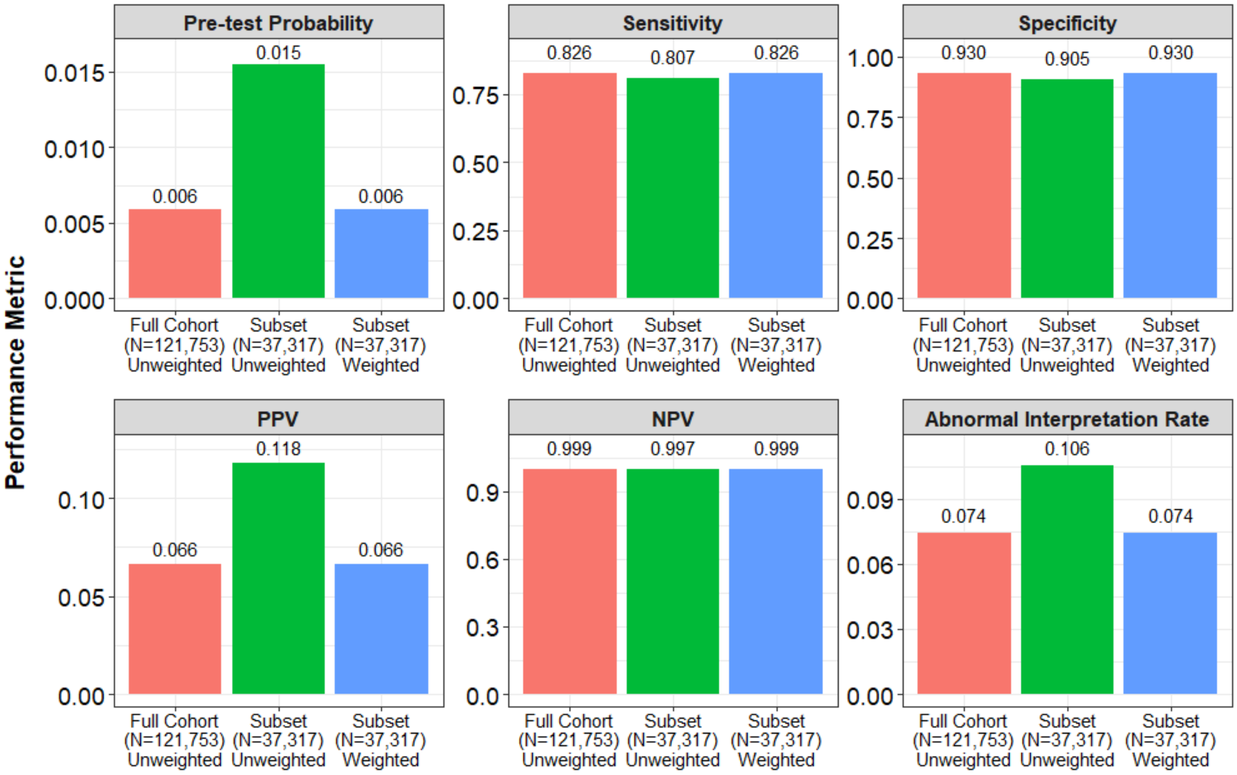

Pre-test probability, sensitivity, specificity, positive predictive value (PPV), negative predictive value (NPV), and abnormal interpretation rate of the radiologist without and with inverse probability weighting. After weighting the performance metrics calculations, the radiologist's estimated performance in the analyzed subset matched the estimates from the full 2D UCLA Health Athena cohort.

© 2022 Hsu W et al. *JAMA Network Open.*

eFigure 4. Architecture of Evaluation Environment

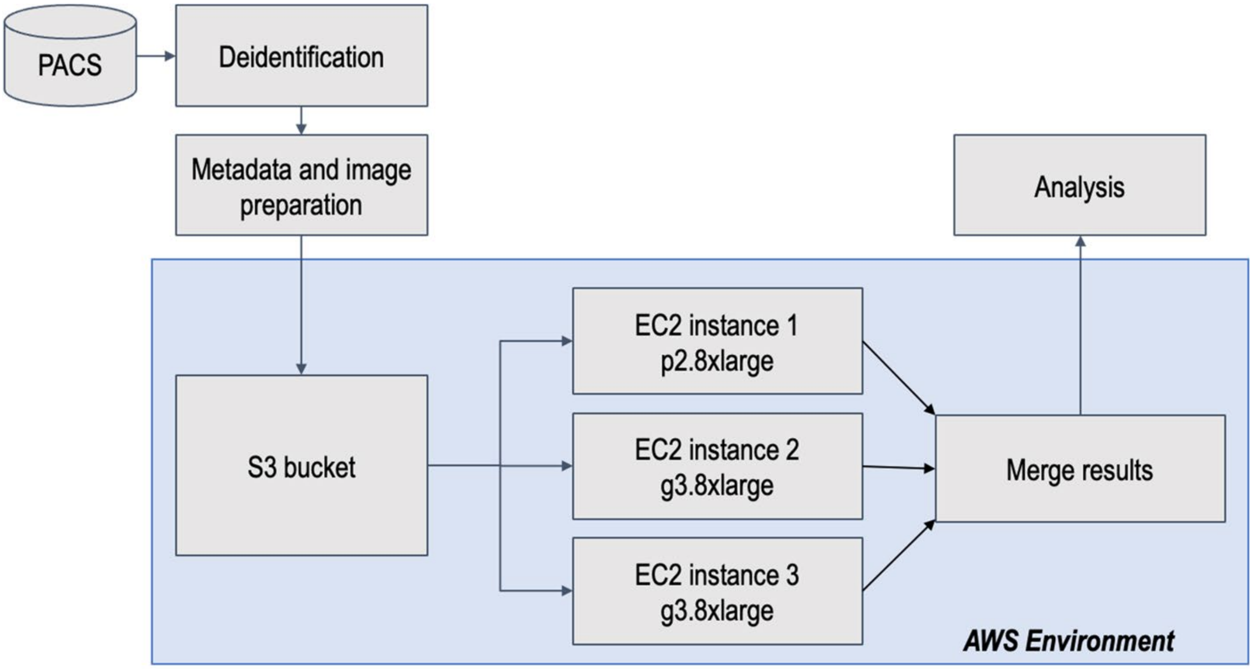

AWS: Amazon Web Services; S3: Simple Storage Service; EC2: Elastic Compute Cloud

© 2022 Hsu W et al. *JAMA Network Open.*

eFigure 5. Histograms of Model Predictions

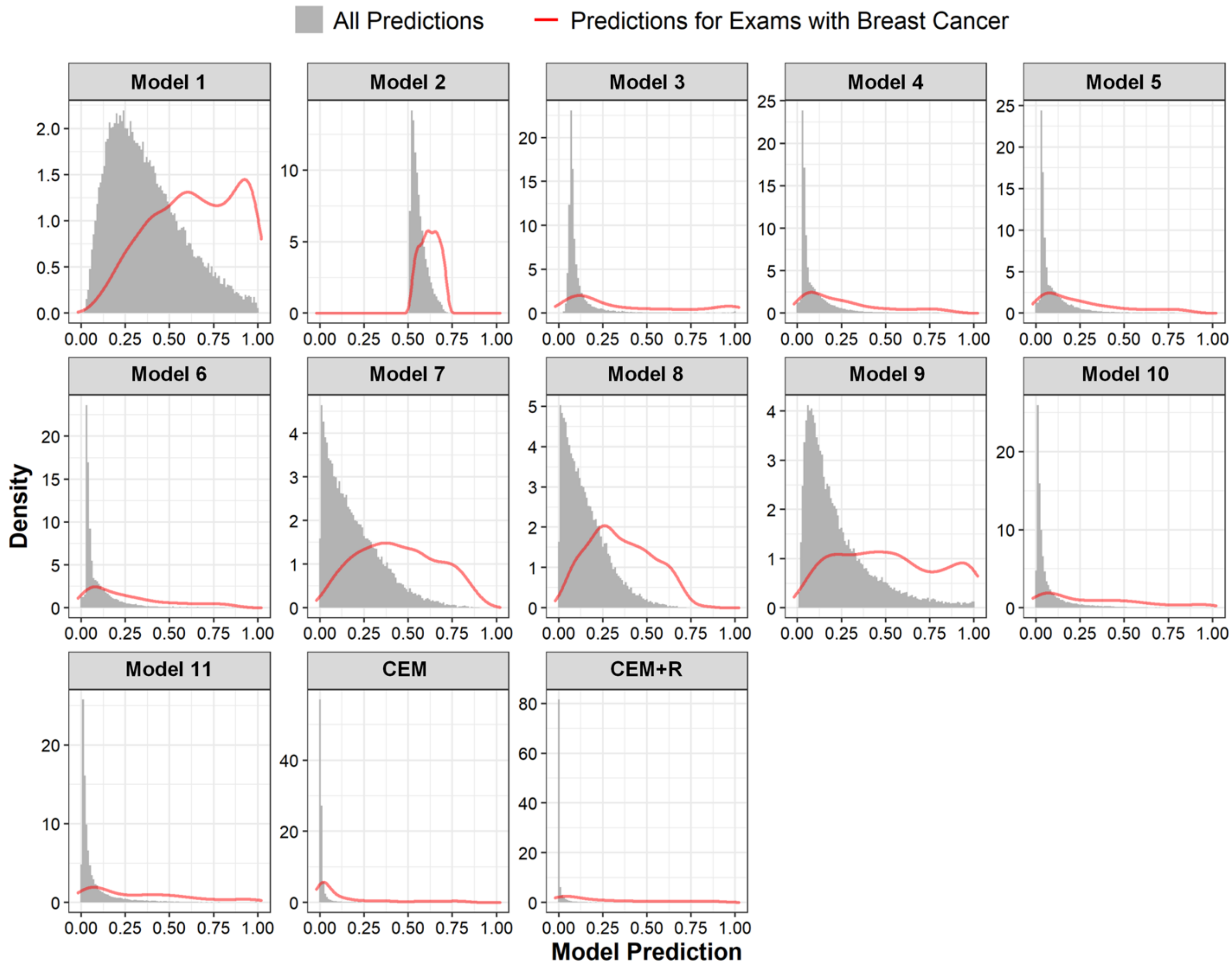

Predictions at the exam-level for each individual model, CEM, and CEM+R are shown. The gray bars show the density of all predictions. The red curves are smoothed density estimates of the model predictions for exams with a breast cancer outcome.

© 2022 Hsu W et al. *JAMA Network Open.*

eFigure 6. Calibration Performance of Challenge Ensemble Method With Radiologist Models By Clinical Characteristic

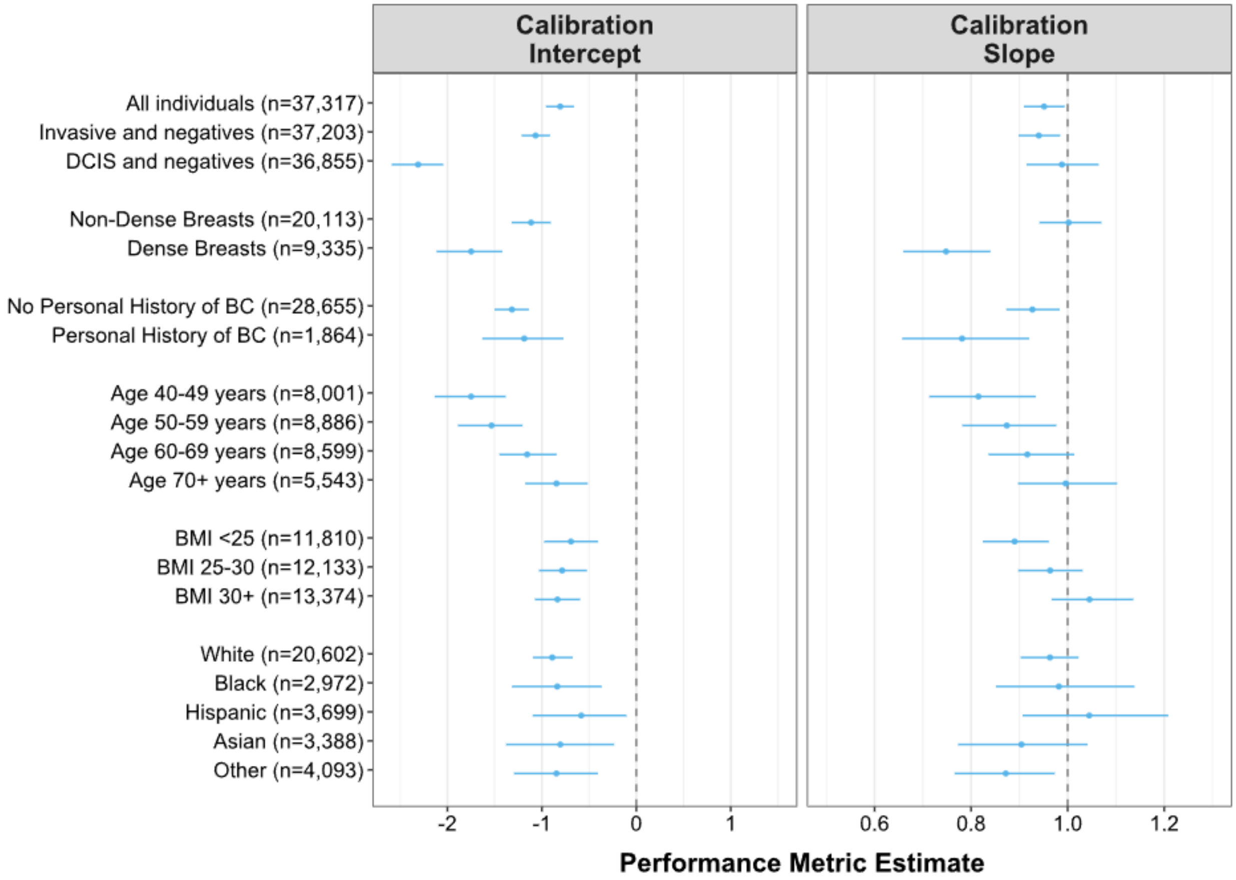

Error bars indicate 95% confidence intervals. Ideal calibration occurs when the intercept is zero and the slope is one, indicated by the vertical dashed lines.

© 2022 Hsu W et al. *JAMA Network Open.*

eReferences

1. Schaffter T, Buist DSM, Lee CI, et al. Evaluation of Combined Artificial Intelligence and Radiologist Assessment to Interpret Screening Mammograms. *JAMA Netw Open*. Mar 2 2020;3(3):e200265. doi:10.1001/jamanetworkopen.2020.0265
2. Sage Bionetworks. DigitalMammographyEnsemble. Accessed 2022-05-01, https://github.com/Sage-Bionetworks/DigitalMammographyEnsemble
3. Sage Bionetworks. The Digital Mammography DREAM Challenge. Accessed 2022-05-01, https://www.synapse.org/#!Synapse:syn4224222
